# Predictors and outcome of cardiac arrest in paediatric patients presenting to emergency medicine department of tertiary hospitals in Tanzania

**DOI:** 10.1186/s12873-022-00679-5

**Published:** 2022-07-12

**Authors:** Amne O. Yussuf, Said S. Kilindimo, Hendry R. Sawe, Elishah N. Premji, Hussein K. Manji, Alphonce N. Simbila, Juma A. Mfinanga, Ellen J. Weber

**Affiliations:** 1 Emergency Medicine Department, Muhimbili University of Health and Allied Science, P.O. Box 65001, Dar es Salaam, Tanzania; 2 Emergency Medicine Department, Muhimbili National Hospital, Dar es Salaam, Tanzania; 3 Department of Emergency Medicine, University of California, San Francisco, CA USA

**Keywords:** Paediatrics, Cardiac arrest, In-hospital cardiac arrest, LMIC, Emergency Department, Tanzania

## Abstract

**Background:**

The survival of children who suffer cardiac arrest is poor. This study aimed to determine the predictors and outcome of cardiac arrest in paediatric patients presenting to an emergency department of a tertiary hospital in Tanzania.

**Methodology:**

This was a prospective cohort study of paediatric patients > 1 month to ≤ 14 years presenting to Emergency Medicine Department of Muhimbili National Hospital (EMD) in Tanzania from September 2019 to January 2020 and triaged as Emergency and Priority. We enrolled consecutive patients during study periods where patients’ demographic and clinical presentation, emergency interventions and outcome were recorded. Logistic regression analysis was performed to identify the predictors of cardiac arrest.

**Results:**

We enrolled 481 patients, 294 (61.1%) were males, and the median age was 2 years [IQR 1–5 years]. Among studied patients, 38 (7.9%) developed cardiac arrest in the EMD, of whom 84.2% were ≤ 5 years. Referred patients were over-represented among those who had an arrest (84.2%). The majority 33 (86.8%) of those who developed cardiac arrest died. Compromised circulation on primary survey (OR 5.9 (95% CI 2.1–16.6)), bradycardia for age on arrival (OR 20.0 (CI 1.6–249.3)), hyperkalemia (OR 8.2 (95% CI 1.4–47.7)), elevated lactate levels > 2 mmol/L (OR 5.2 (95% CI 1.4–19.7)), oxygen therapy requirement (OR 5.9 (95% CI 1.3–26.1)) and intubation within the EMD (OR 4.8 (95% CI 1.3–17.6)) were independent predictors of cardiac arrest.

**Conclusion:**

Thirty-eight children developed cardiac arrest in the EMD, with a very high mortality. Those who arrested were more likely to present with signs of hypoxia, shock and acidosis, which suggest they were at later stage in their illness. Outcomes can be improved by strengthening the pre-referral care and providing timely critical management to prevent cardiac arrest.

**Supplementary Information:**

The online version contains supplementary material available at 10.1186/s12873-022-00679-5.

## Background

Paediatric cardiac arrest has a high mortality rate and of those who survive, outcomes are often poor, with disability and long-term health care dependence [1].

In high-income countries (HICs) such as the United States (US), nearly 6,000 children receive in-hospital cardiopulmonary resuscitation (CPR) each year (approximately 0.3% of all hospitalized children) and generally have positive outcomes [2, 3]. In contrast, studies in Sub Saharan Africa have shown that paediatric cardiac arrest in the emergency department ranges between 4.1% and 28% [1, 4].

Recognition of early predictors of cardiac arrest can allow emergency physicians to rapidly identify a child who is at risk of developing cardiac arrest and intervene to prevent it. Several studies in high income countries have looked at predictors of arrest [1, 3, 5], but these may not be relevant in Low- and Middle-Income Countries (LMICs) where diseases are different, time to care is longer and health care systems and resources are fewer.

Since the incidence of cardiac arrest in LMICs is higher than in other settings, the predictors may be different [1, 6]. Nevertheless, there is scarcity of data on the predictors of paediatric cardiac arrest in our setting. Emergency Medicine is a relatively new field in Sub Saharan Africa and studies on such predictors have not previously been performed.

Therefore, this study aimed to determine the predictors and outcome of cardiac arrest at the Emergency Medicine Department of Muhimbili National Hospital (EMD) in Tanzania.

## Methodology

### Study design

This was a prospective cohort study of all critical paediatric patients presenting to the EMD from September 2019 to January 2020.

### Study setting

This study was conducted at the EMD at Muhimbili National Hospital (MNH) in Dar es salaam, Tanzania. MNH is a public, tertiary level hospital in the country. The hospital has a full capacity EMD that receives referrals from across the country, as well as serving as an immediate tertiary referral facility for Dar es Salaam. The EMD is the only primary training site for Emergency Medicine (EM) residency training in the country. It is staffed with emergency physicians, residents in an emergency medicine program, medical officers and critical care nurses. The EMD provides emergency care and resuscitation, serving an average of 200 patients a day; approximately one quarter of them are children under 18 years who have gone through multiple levels of care before arriving to the EMD.

### Study participants

Paediatric patients aged 28 days to 14 years, triaged as emergency and priority category (equivalent to Emergency Severity Index (ESI) levels 1, 2 and 3, respectively) presenting to the EMD were eligible. ESI is a five-level emergency department triage algorithm that categorizes patients by evaluating the acuity of illness and the resources needed to manage the patient. We collected data on alternate days among consecutive paediatric patients attending EMD. The study was approved by Muhimbili University of Health and Allied Sciences (MUHAS) Institutional Review Board and MNH and parents/guardians provided written consent.

### Study protocol

We used a standardized structured case report form to document demographic information, clinical presentation, including results of the physician’s primary survey (compromised airway, breathing or circulation) vital signs, investigations and results, provisional diagnoses, EMD interventions and outcome for all eligible patients, using providers’ and nurses’ clinical notes and observing interventions. Paediatric and advanced life support chart parameters were used as a reference for tachypnoea, tachycardia and bradycardia in different age groups. For patients who arrested, we determined duration of CPR, duration to ROSC and 24-h cardiac arrest outcome for those who attained ROSC. All the investigations and treatment given to patients were based on the provider’s decision.

### Outcomes

The primary outcome was paediatric cardiac arrest in the EMD. The secondary outcome was the to provide a descriptive analysis of outcome of paediatric cardiac arrest in the EMD. The sample size estimation was based on a pilot study conducted at EMD MNH from 1^st^ January to 31^st^ March 2019 using age as a predictor of cardiac arrest. Sample size was calculated using the proportion of subjects with age group less than 5 years and 5 years and above (0.55 and 0.45 respectively) and the proportion of subjects who develop cardiac arrest with age group less than 5 years and 5 years and above (0.081 and 0.021 respectively). Using an alpha of 0.5 and power of 0.80, the minimum sample size required was 476 patients.

### Data analysis

Data from the CRF was entered into REDCap (version 7.2.2, Vanderbilt, Nashville, TN, USA), imported to Microsoft Excel 2016 and transferred into the Statistical Package for Social Science (version 25.0, IBM, LTD, North Carolina, USA) for analysis. Because the goal of the study was to determine the predictors and outcome of cardiac arrest, patients’ characteristics, initial presentations and management given prior to arrest were considered as covariates. Standard descriptive statistics were reported using median and interquartile range for continuous variables and frequency and percentage for categorical variables.

Variables were age, sex, referral status, length of stay at the referring facility, airway abnormality (patients with airway compromise in primary survey), breathing abnormality (head nodding, nasal flaring, lower chest wall indrawing and the use of accessory muscles), circulatory abnormality (patients with cyanosis, delayed capillary refill and cool/cold extremities), tachycardia, bradycardia, tachypnoea, bradypnea, hypoxia, investigations and management given prior to arrest. To determine the association between candidate variables and risk of developing cardiac arrest, a univariate regression analysis was conducted. Variables associated with cardiac arrest with a *p* value of < 0.2 were then included in multivariate logistic regression. Duration of CPR was reported with median and interquartile ranges.

## Results

**Fig. 1:**
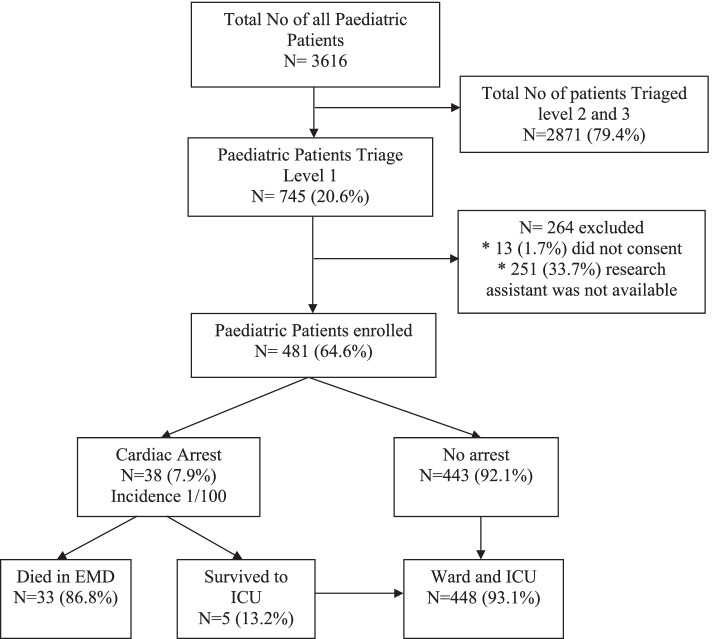
Flow of paediatric patients at EMD-MNH

During the study period, 3616 paediatric patients presented to the EMD and 745 (20.6%) were seen as emergency and priority. 481(64.6%) consented to be enrolled in the study (Fig. 1).

The overall median age was 2 years [IQR of 1–5 years], where by 372 (77.3%) were ≤ 5 years, 109 (22.7%) were > 5 years. 294 (61.1%) were males. The majority of children (69%) were referred from other health facilities with overall median length of stay at referring facility 2 days [IQR 1–3 days]. On the primary survey, 48.9% of patients had abnormal breathing and 27.9% had abnormal circulation. Tachycardia (for age) was the most frequently reported (46.4%) abnormal vital sign (Table 1).

**Table 1:** Demographic and clinical characteristics of paediatric patients triaged emergency and priority who presented to EMD-MNH

VARIABLES	ARREST, n (%) *N* = 38	NO ARREST, n (%) *N* = 443
**Age**		
**Median age [IQR]: 2 years [1–5 years]**	**1 year [0-1 year]**	**2 years [1–5 years]**
≤ 5 years	32 (8.6)	340 (91.4)
> 5 years	6 (5.5)	103 (94.5)
**Sex**		
Female	23 (12.3)	164 (87.7)
**Referral status**		
Referred	32 (9.6)	301 (90.4)
**Primary survey**		
Airway abnormal	16 (28.1)	41 (71.9)
Breathing abnormal	25 (10.6)	210 (89.4)
Circulation abnormal	27 (20.1)	107 (79.9)
**Initial Vital signs**		
Tachycardia for age	16 (7.2)	207 (92.8)
Bradycardia for age	6 (66.7)	3 (33.3)
Tachypnea for age	24 (11.8)	179 (88.2)
Axillary Temperature > 37.5 ^0^C	16 (0.03)	152 (31.6)
Altered mental status	38 (23.9)	121 (76.1)
Hypoxia (Spo2 < 94%)	17 (16.3)	87 (83.7)

**Fig. 2:**
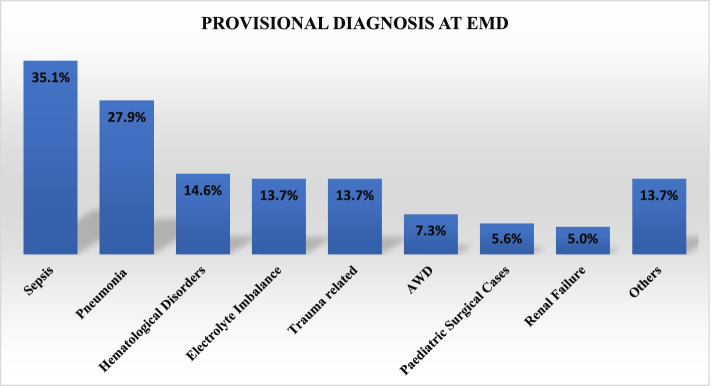
Provisional Diagnosis at EMD-MNH, Dar es salaam Tanzania. **Others (Seizure disorders, Heart failure, Severe Acute Malnutrition, Diabetic Ketoacidosis, Malignancy, Steven Johnson Syndrome). ** AWD – Acute Watery Diarrhoea. NB: One child could have more than 1 diagnosis

In the entire cohort, sepsis (35.1%) was the most frequently reported provisional EMD diagnosis followed by pneumonia (27.9%) (Fig. 2). Among all children, 65.5% were acidotic, 5.3% had hyperkalemia and 55.7% had elevated lactate levels. More than half of patients received antibiotics and intravenous crystalloid fluids. Over a third of patients required oxygen therapy and less than 10% were intubated. (Table 2)

**Table 2:** Management of paediatric patients triaged emergency and priority who presented to EMD-MNH

VARIABLES	FREQUENCY (%)
**Investigations (abnormal parameters)**	
pH < 7.35	201/307 (65.5)
pH > 7.45	71/307 (23.1)
K < 3.5 mmol/L	133/374 (35.6)
K + > 5.5 mmol/L	20/374 (5.3)
**Lactate ≥ 2 mmol/L**	**171/307 (55.7)**
**Treatment given pre-arrest**	
Antibiotics	324 (67.4)
Intravenous crystalloid fluids	318 (66.1)
Oxygen therapy	185 (38.5)
Blood transfusion	63 (13.1)
Intubation	43(8.9)

Overall, 38 (7.9%) patients developed cardiac arrest in the EMD, of whom the majority were ≤ 5 years 32 (84.2%) and referred from other health care facilities 32 (84.2%). Patients who were referred from other health care facilities, patients with compromised airway and breathing who arrived hypoxic and needed oxygen therapy and intubation were significantly more likely to have a cardiac arrest. Moreover, patients with bradycardia on arrival and with compromised circulation in primary survey also predicted cardiac arrest, while patients who received antibiotics were found to have a reduced risk of cardiac arrest in EMD (Table **3**).

**Table 3:** Predictors of cardiac arrest in paediatric patients triaged emergency and priority at EMD-MNH

VARIABLE	Multivariate OR (95% CI)	*P* value
**Primary survey**		
Circulation normal		
Circulation abnormal	5.9 (2.1—16.6)	0.001
**Initial vital signs**		
Bradycardia for age	20.0 (1.6—249.3)	0.005
**Investigations**		
K + 3.5–5.5 mmol/L		
K + > 5.5 mmol/L	8.2 (1.4–47.7)	0.026
Lactate < 2 mmol/L		
Lactate ≥ 2 mmol/L	5.2 (1.4–19.7)	0.012
**Treatment given pre-arrest**		
No oxygen therapy		
Need for oxygen therapy	5.9 (1.3–26.1)	0.015
No intubation		
Need for intubation	4.8 (1.3 – 17.6)	0.01

^*^We only did multivariate analysis for those *p*-value ≤ 0.2

In multivariate regression only circulation abnormalities on primary survey, bradycardia, hyperkalemia, elevated lactate levels and need for oxygen therapy and intubation were independently associated with cardiac arrest in the EMD, whereas receiving antibiotics was associated with a reduced risk of cardiac arrest in the EMD. (Table 3) Age was not found to be an independent predictor in the multivariate regression.

### Outcome of cardiac arrest in paediatric patients who presented to EMD-MNH 

Among 38 children who had cardiac arrest, 33 (86.8%) died while in EMD. Only 5 (13.2%) attained ROSC and survived to ICU admission. The median CPR duration was 30 min [IQR 20–38 min] overall, while in those who achieved ROSC, the median duration of CPR was 11 min [IQR 10–13 min]. Most patients who died were ≤ 5yrs (84.2%) or referred from other health care facilities (84.2%). Among those referred, 44.7% spent more than 24 h at the referring facility, 24 h mortality was 35 (92%), with only 3 patients surviving more than 24 h. (Table 4).

**Table 4:** Outcome of cardiac arrest in paediatric patients triaged as emergency and priority at EMD-MNH

	**Median [IQR]**	**Number n (%)**
Median CPR duration (minutes) [IQR]	30 min [20–38 min]	
**Cardiac Arrest**		**38 (7.9%)**
Died in EMD		33 (86.8)
ROSC at EMD		5 (13.2)
Median duration to ROSC (minutes) [IQR]	11 min [10–13 min]	
ICU admission		5(13.2)
24 h mortality (including died in ED)		35 (92.0)

## Discussion

In this study, we aimed to identify the predictors and outcome of cardiac arrest in paediatric patients presenting to a tertiary emergency medicine department in a LMIC. Patients with compromised circulation, bradycardia as the initial vital sign, hyperkalemia, elevated lactate levels, patients who arrived at a state of requiring oxygen therapy and intubation were the independent predictors of cardiac arrest while in the EMD. The incidence of paediatric cardiac arrest is higher than previously documented in-hospital cardiac arrest in high income countries [7, 8]. Among those who developed cardiac arrest, only 13% survived to ICU admission, which emphasizes the importance of recognizing these predictors before the arrest occurs.

In this study, bradycardia as the initial vital sign was found to be independently associated with cardiac arrest. A multicenter cohort study done in the US also found that paediatric patients with initial bradycardia had a higher likelihood of developing cardiac arrest in the EMD [9]. Bradycardia in children is known to be an ominous sign, usually associated with hypoxia and imminent cardiac arrest. This study confirms the need for early, aggressive resuscitation in children presenting with bradycardia.

In LMIC, most EMD have only a few blood-pressure cuffs for children. BP is rarely measured for young children; therefore, assessment of circulation is mainly done by evaluating the skin and mucous membranes together with the capillary refill time. Using this simple clinical assessment to determine adequacy of circulation during the primary survey, we found compromised circulation was independently associated with cardiac arrest.

Elevated blood lactate levels provide an insight into the presence of impaired tissue perfusion [10]. Lactate has been found to be a useful predictor in identifying critically ill children at high risk of death in the emergency and paediatric intensive care settings but its utility in LMICs EMDs is not as well studied [11]. We found lactate (> 2 mmol/L), done at the point of care, to be an independent predictor of cardiac arrest, suggesting it is a useful addition to physical exam. Moreover, the presence of an elevated lactate in over half of all of our patients suggests that the patients are in very late stages of disease even when they first arrive at our facility. This may be due to delayed care seeking or delays at outside facilities.

Hyperkalemia was also found to be independently associated with cardiac arrest. A previous study also noted that a high potassium level was more likely to be associated with bradycardia, reduced urine output and acidosis [12]. This combination of abnormalities signals not only an increased likelihood of cardiac arrest but also a decreased likelihood of survival [12].

Critically ill children who were hypoxic and arrived at a state of requiring oxygen therapy and intubation were significantly more likely to have a cardiac arrest. Patients who are intubated in the EMD are some of the most critically ill patients and carry a significant risk of deterioration if not intervened early. Due to the lack of sufficient ventilators and ICU beds in our setting, only the sickest children undergo intubation. Therefore, intubation is done at a very late stage in their stay at the EMD and thus carries a high risk of cardiac arrest. Similar to other studies, intubation was found to be independently associated with cardiac arrest [9, 13].

Prior studies in HICs have found that age is an important predictor of paediatric cardiac arrest where by children ≤ 5 years old are more vulnerable [14]. Literature from LMICs also suggests that the highest mortality rate in children who come to the EMD is age ≤ 5 years. In our findings, the majority of paediatric patients who developed cardiac arrest were ≤ 5 years but the difference in age was not significant between the two groups in the regression analysis. One possible explanation for this could be that the presence of other stronger risk factors masked the effect of age as a predictor of arrest.

In our study, over two-thirds of all critically ill patients were referred from other health care facilities. More patients who arrested had been referred and referral was associated with occurrence of cardiac arrest in univariate analysis, but was not an independent predictor in multivariate analysis. We suspect this is due to the fact that referral patients are more likely to critically ill and would arrive with risk factors that were shown to be independent predictors of arrest: circulatory compromise, abnormal vital signs, acidosis and need for intervention. Similar findings were obtained in a retrospective study done in Cincinnati which found that referred patients were significantly more likely to have greater severity of illness [15]. Thus delays caused by hierarchal system of referring patients could also be a contributing factor to a relatively high proportion of arrests in our EMD. Therefore, strengthening of the pre-referral care and early referral of these critically ill patients is important so as to prevent cardiac arrest.

The median duration for CPR in these children was 30 min, but in the small proportion who achieved ROSC, it was 11 min. CPR lasting ≤ 5 min was found to be one of the most important prognostic factors affecting outcome of cardiopulmonary arrest in the EMD [16]. Prolonged resuscitation of more than 15 min was associated with poor outcome during CPR [17]. This suggests that prolonged CPR in the EMD is rarely effective, and duration should be considered in determining when to stop resuscitative efforts.

### Limitations

This was a single center study at a tertiary hospital. Patients at our facility may therefore have been sicker and more prone to cardiac arrest; however, it is also possible that some with cardiac arrest never reached our department. However, we see no reason to believe that the risk factors (other than perhaps referral) would be different. Also, patients were enrolled on alternate days so not all arrests were considered. Not all patients had all tests, due to the lack of availability and provider decisions.

## Conclusion

In-hospital paediatric cardiac arrest is not infrequent in our setting, and mortality is high after arrest. Immediate recognition of compromised circulation, bradycardia, hyperkalemia, elevated lactate levels, as well as the need for oxygen therapy and intubation in children signify high risk for cardiac arrest with a need for time critical management to prevent arrest and death. This study suggests the need for the use of a warning system and clinical protocols in the EMD, and strengthening pre-referral treatment so as to provide better and more timely care of critically ill children. Research interventions aimed to identify and treat children at risk of cardiac arrest within the resource constraints of this setting are also needed.

## Supplementary Information

**Additional file 1: Supplemental Table 1.**Univariate analysis of predictors of cardiac arrest in paediatric patientstriaged emergency and priority at EMD-MNH.

## Data Availability

The dataset supporting the conclusion of this article is available from the authors on request.
