# Supplementary material for: Predictors and outcome of cardiac arrest in paediatric patients presenting to emergency medicine department of tertiary hospitals in Tanzania
Source: BMC Emerg Med. 2022 Jul 12;22:126. doi: 10.1186/s12873-022-00679-5 (PMC9277961; doi:10.1186/s12873-022-00679-5)
Supplement: Supplementary file 1 — Additional file 1: Supplemental Table 1.Univariate analysis of predictors of cardiac arrest in paediatric patientstriaged emergency and priority at EMD-MNH. [file 12873_2022_679_MOESM1_ESM.docx]

**Supplemental Table 1: Univariate analysis of predictors of cardiac arrest in paediatric patients triaged emergency and priority at EMD-MNH**

|  | **VARIABLES** | **Arrest**  **n=38** | **No arrest**  **n=443** | **Univariate**  **0R (95% CI)** | **P value** |  |
| --- | --- | --- | --- | --- | --- | --- |
| **Age** | > 5 years  ≤ 5 years | 6 (5.5%)  32 (8.6) | 103 (94.5%)  340 (91.4) | Reference  1.6(0.7-4.0) | 0.296 |  |
| **Sex** | Male  Female | 15 (5.1)  23 (12.3) | 279 (94.9)  164 (87.7) | Reference  2.6(1.3-5.1) | 0.006 | |
| **Referral status** | Not Referred  Referred | 6 (4.1%)  32 (9.6) | 142 (95.9)  301(90.4) | Reference  2.5(1.1-6.1) | 0.043 |  |
| **Length of stay at referring hospital** | < 24 hours  >24hours | 3 (8.8)  29 (9.7) | 31 (91.2)  270 (90.3) | Reference  1.1(0.3-3.9) | 0.870 |  |
| **Primary survey** | Airway normal  Airway abnormal  Breathing normal  Breathing abnormal  Circulation normal  Circulation abnormal | 16 (28.1)  25 (10.6)  27 (20.1) | 41 (71.9)  210 (89.4)  107 (79.9) | Reference  7.1(3.5-14.6)  Reference  2.1(1.1-4.3)  Reference  7.7(3.7-16.1) | 0.001  0.030  0.001 |  |
| **Initial vital sign** | Normal respiratory rate for age  Bradycardia for age  Tachypnoea for age  Normal saturation of oxygen  Hypoxia (SpO2 <94%) | 24 (11.8)  17 (16.3) | 179 (88.2)  87 (83.7) | Reference  27.5(6.6-115.1)  2.5(1.3-5.0)  Reference  3.3(1.7-6.5) | 0.001  0.01  0.001 |  |
| **ED Provisional diagnosis** | No Sepsis  Sepsis  No respiratory failure  Respiratory failure | 20 (11.8)  9 (37.5) | 149 (88.2)  15 (62.5) | Reference  2.2(1.1-4.2)  Reference  8.8(3.6-22.0) | 0.021  0.001 |  |
| **Investigations** | pH ≥7.35  pH< 7.35  K+ 3.5-5.5 mmol/L  K+ >5.5 mmol/L  Lactate < 2mmol/L  Lactate ≥ 2mmol/L | 35 (17.4)  5 (25.0)  31 (18.1) | 166 (82.6)  15 (75.0)  140 (81.9) | Reference  7.4(2.2-24.5)  Reference  3.2(1.1-9.5)  Reference  4.1 (1.7-9.6) | 0.001  0.032  0.001 |  |
| **Pre arrest treatment given** | No oxygen therapies  Need for oxygen therapy  No intubation within EMD  Need for intubation within EMD  No Intravenous crystalloid fluids given  Intravenous crystalloid fluids given  No antibiotics given  Antibiotics given | 31 (16.8)  16 (37.2)  29 (9.1)  18 (5.6) | 154 (83.2)  27 (62.8)  289 (90.9)  306 (94.4) | Reference  8.3 (3.6-19.3)  Reference  11.2 (5.3-23.8)  Reference  1.7 (0.8-3.7)  Reference  0.4 (0.2-0.8) | 0.001  0.001  0.20  0.008 |  |
